# Supplementary material for: Atomic intercalation to measure adhesion of graphene on graphite
Source: Nat Commun. 2016 Oct 31;7:13263. doi: 10.1038/ncomms13263 (PMC5095517; doi:10.1038/ncomms13263)
Supplement: Supplementary Information — Supplementary Figures 1-6, Supplementary Notes 1-3 and Supplementary References. [file ncomms13263-s1.pdf]

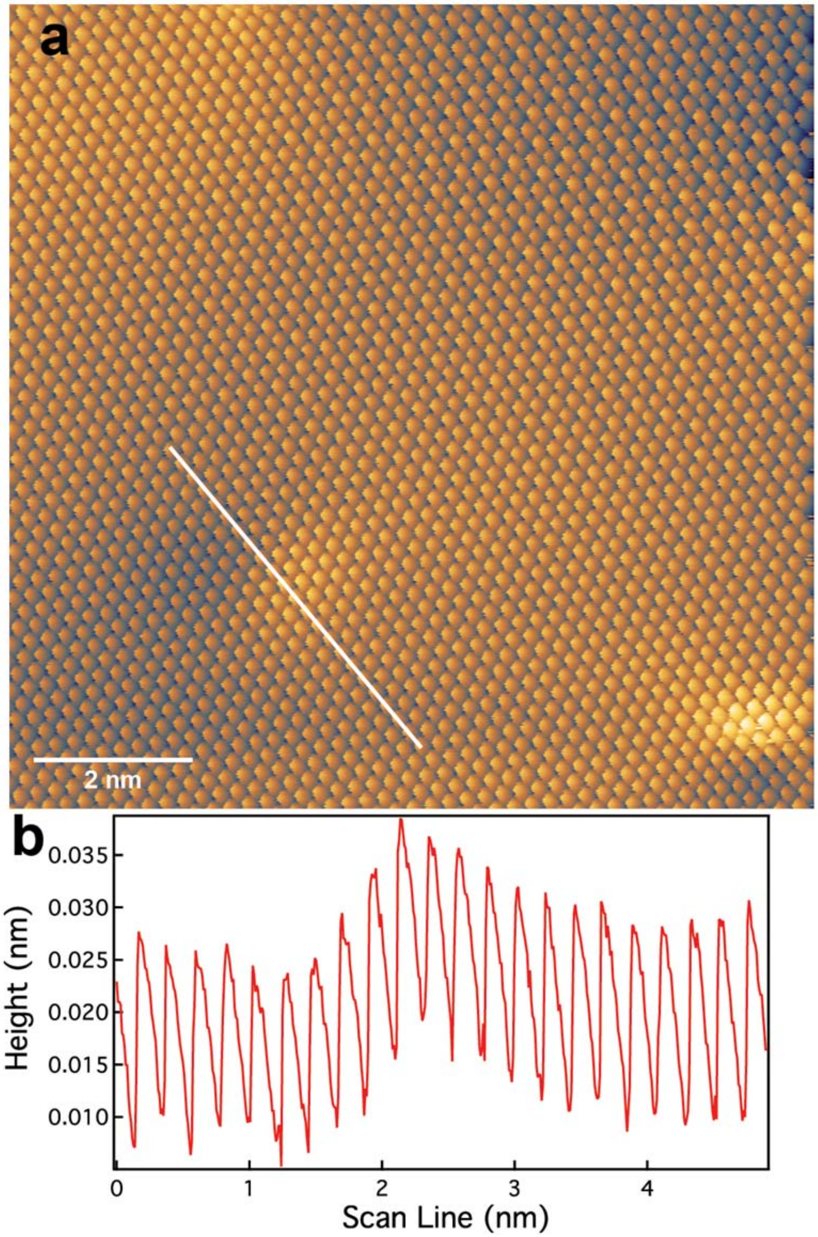

Supplementary Figure 1 | Small size blisters possibly caused by deeper Ne intercalates. (a) STM image (U_{sample} = 0.1 V, I_t = 0.15 nA, T = 4.3 K) captures two small protrusions on the surface of Ne intercalated graphite: one is indicated with a line across, the other is the brighter spot in the lower right corner. (b) The corresponding line profile for the scan line in (a) across the small blister.

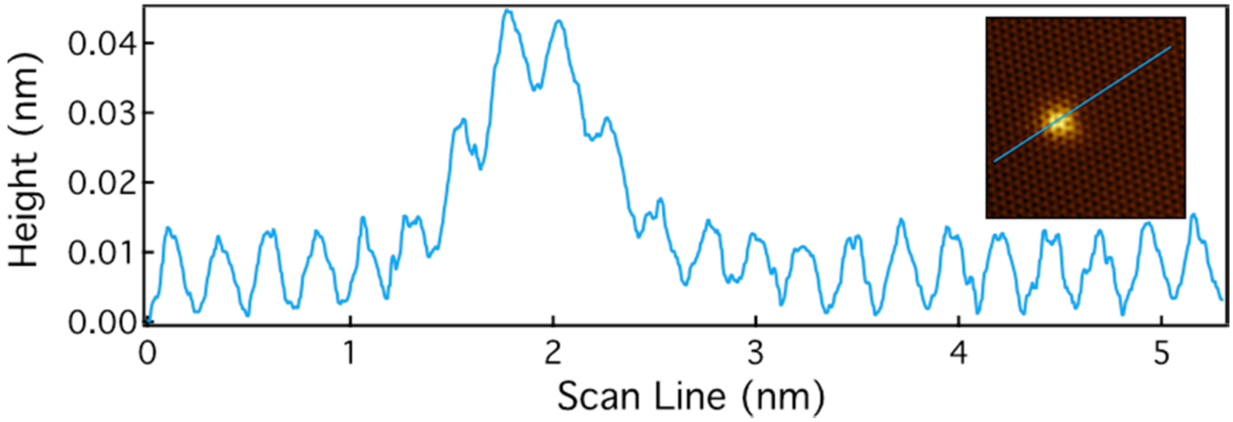

Supplementary Figure 2 | A representative cross-section across the blister in Figure 1c. The blister has a smaller height of ~ 0.03 nm, while the graphite lattice corrugation is also scaled down, ~ 0.1 Å.

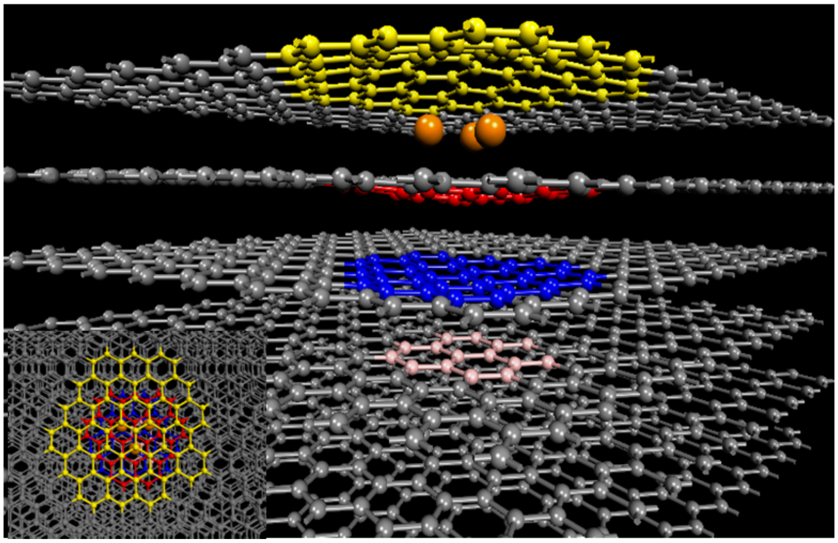

Supplementary Figure 3 | A model of Ne intercalates in a 12x12 6-layer graphite slab determined by DFT calculations. In this configuration, three Ne atoms (colored in orange) are intercalated between the 1st and 2nd layers. The colored carbon atoms in different layers correspond to the deformed atoms caused by the Ne intercalates, as oppose to the gray C atoms without deformation. The main figure provides a lateral view of the atomic configuration while a top view of the same configuration is given in the inset figure.

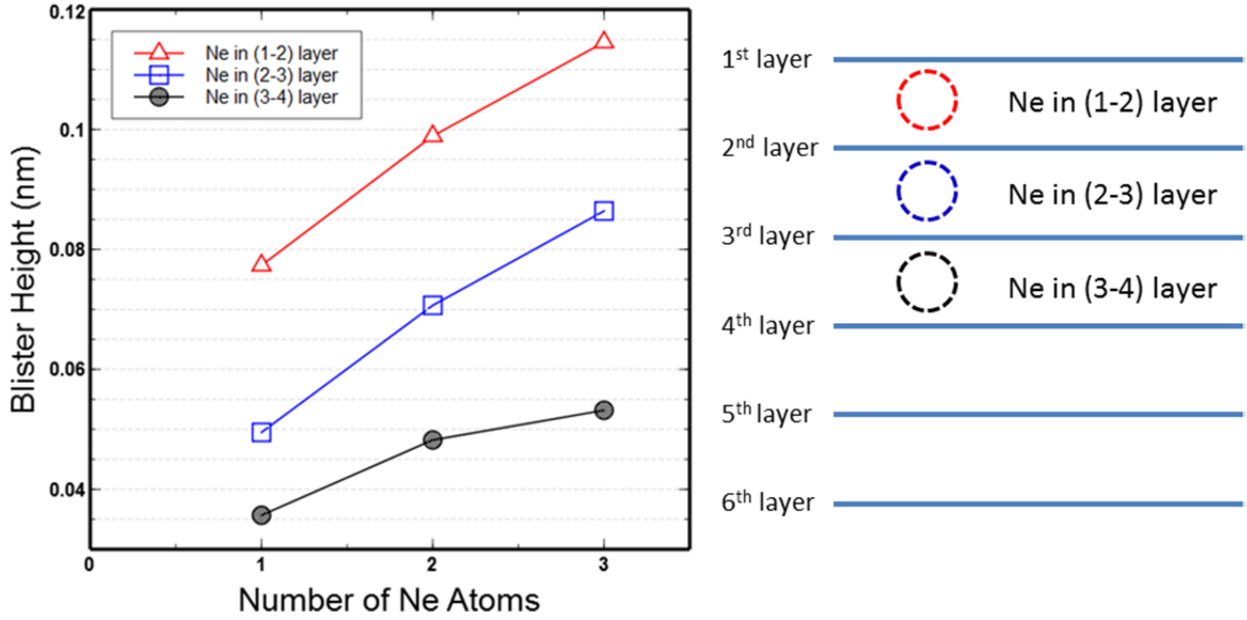

Supplementary Figure 4 | Blister height for different intercalation depths (measured from the top layer deformation of HOPG as obtained from DFT calculations) and as a function of the number of Ne atoms (left panel). The red triangles represent the case when Ne atoms are located between the top (1st) and 2nd graphene sheets, the blue squares represent the case when Ne atoms are positioned between the 2nd and 3rd sheets, and the black circles represent the case when Ne are between the 3rd and 4th sheets. The deeper level of intercalates leads to smaller heights of the blister in the top surface layer.

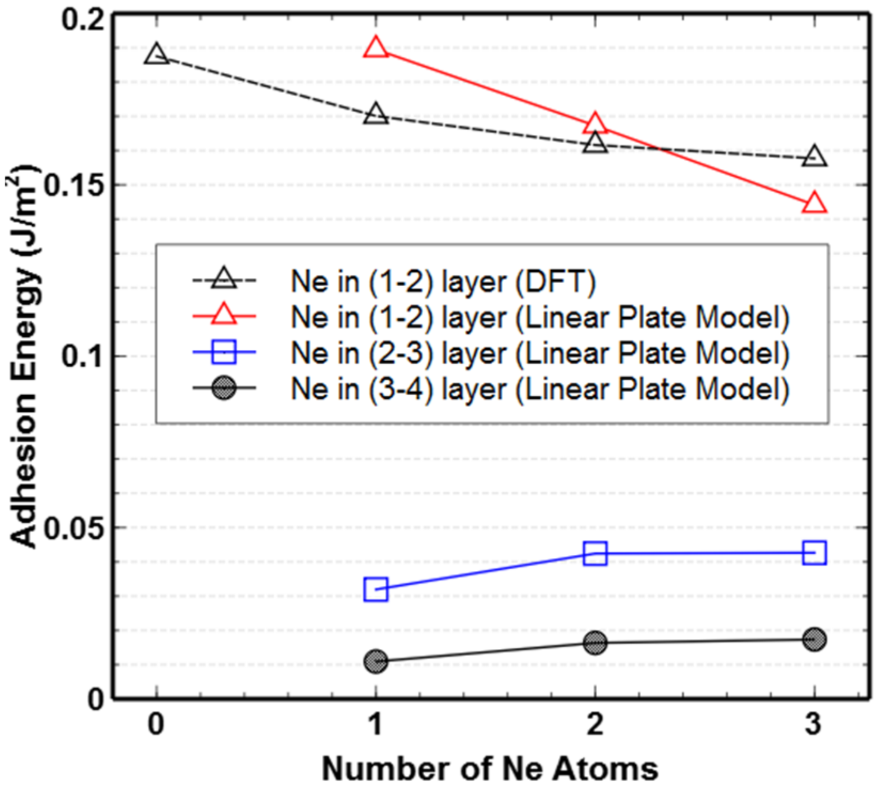

Supplementary Figure 5 | The adhesion energy (derived from the linear plate model for the top graphene sheet deformation as obtained in DFT models) as a function of the number of Ne atoms. The red triangles represent the case when Ne is located between the top and 2nd graphene sheets, the blue squares represent the case when Ne is positioned between the 2nd and 3rd sheets, while the black circles represent the case when Ne is between the 3rd and 4th sheets. The adhesion energy is in close agreement to the DFT results (black triangles connected with dashed lines) and macroscopic values only for the case of sub-surface intercalates.

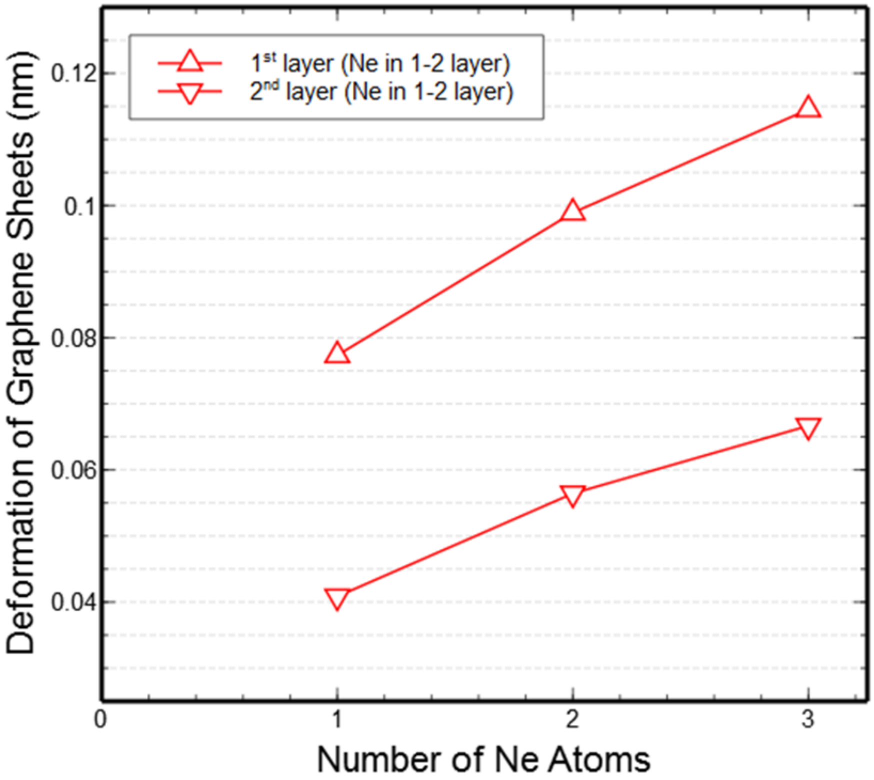

Supplementary Figure 6 | Maximum deformation of graphene sheets encapsulating the Ne atoms as a function of the number of Ne atoms. The immediate layers below the Ne are always deformed, although the deformation is much higher for the open surface.

Supplementary Note 1: "Triangular" blister

The "triangular" shape mentioned in the main text is used for easy identification. To quantify the shape of such blisters, we approximated them as having a Gaussian curvature, and then by averaging over all measured radii. In addition, the "triangular" shaped blister shown in Figure 1c and in Supplementary Figure 2 is not likely to be associated with deeper Ne intercalates, since the constant-current topographic corrugation of the graphite lattice (individual atom) is also scaled down. When the STM topography is appropriately rescaled, so that the corrugation of the unperturbed graphite lattice matches between Figures 1b and 1c, the shape and cohesion energy obtained from blisters in Figures 1b and 1c become closely comparable. Our main point of including this data is to show that one should be aware of "artifacts" of STM topography, mostly due to unknown electronic state of the tip. By normalizing to a certain chosen corrugation may be a way to account for these artifacts. For comparison, we have included additional experimental data on blisters with possible deeper Ne intercalation in Supplementary Figure 1. In this case the height of the blister is significantly smaller, while the topographic corrugations of graphite lattice are comparable between Figure 1b, Figure 5 and Supplementary Figure 1.

Supplementary Note 2: Analysis of deeper layers Ne intercalation

With regard to the possibility of deeper layers Ne intercalation, we carried out additional experiments and DFT calculations.

DFT calculations were carried out for Ne atoms intercalated between 1st and 2nd, 2nd and 3rd, 3rd and 4th layers of a 6-layer slab of graphite with a 12x12 supercell. In order to handle the large size of the system (containing 1728 C atoms) calculations were performed using the Quickstep module of CP2K program.[1,2] The optimized MOLOPT basis set[3] has been used together with Goedecker, Teter and Hutter (GTH) pseudopotentials to represent the core electrons.[4] Standard PBE[5] exchange-correlation functional corrected to include long-range dispersion interactions using Grimme-D3 method[6] was considered for the treatment of electronic exchange and correlation. Analysis of atomic deformations induced by Ne atoms intercalation (see Supplementary Figure 3) was obtained by relaxing all layers of the slab model excepting the bottom layer. The heights of the protrusions caused by the Ne atoms in each graphene layer were measured based on the final relaxed DFT models and are given in Supplementary Figures 4. The height of the blister is substantially reduced when intercalating beyond the first layer. When the linear plate model was applied to the blisters caused by Ne intercalations in different depths, only the blisters from the sub-surface intercalation yield adhesion energy of 0.15-0.20 J m^{-2} (Supplementary Figure 5), which is consistent with DFT calculations (and close to the

macroscopic value). For deeper intercalations the corresponding adhesion energies drop below 0.05 J m^{-2}, due to decreasing curvature of the blister as measured at the top surface. Therefore, for blisters shown in Figure 1b and Figure 5, the assumption of sub-surface intercalation seems to be valid.

Experimentally, we detected occasional small protrusions after Ne^+ sputtering (Supplementary Figure 1), that may correspond to deep intercalates. In Supplementary Figure 1a, we show two blisters with measured heights in the range of ~ 0.02-0.03 nm, an order of magnitude smaller than the ones shown in Figure 1b and Figure 5 (0.1-0.2 nm). Note that the constant-current topographic corrugations of graphite lattice are comparable between Figure 1b, Figure 5 and Supplementary Figure 1, in contrast to the triangular case mentioned above. Given the uncertainty of the exact structure of such possibly deep intercalates, such protrusions are not fit for direct analysis of adhesion energy.

In addition, the calculations show that Ne intercalates distort the neighbor graphene layers encapsulating the Ne atom. However, the layers in the direction of the open graphite surface are distorted 2-3 times (judged by the height) more than those in the direction of the graphite bulk (Supplementary Figure 6). Such deformations on both sides of the Ne intercalate are not accounted for by the plate model and are a source of systematic underestimation of the adhesion energy. However, as seen in Supplementary Figure 5, the error is small and will likely be masked by other experimental errors. At the same time, the trend of reduction of adhesion energy with increasing number of intercalated atoms is well-reproduced by the plate model.

Supplementary Note 3: Temperature dependence

The STM profile measurements over different blisters were performed at 77 K in a highly controlled UHV environment. As a result the information obtained at the atomic scale should be considered trustworthy and furthermore could be directly correlated to the results of DFT simulations performed at 0 K. In addition, our 4 K and 77 K measurements yield consistent parameters for the blister. In a recent paper[7] by using molecular dynamics simulations to investigate the temperature dependent adhesion of a suspended graphene sheet on a trench of 15 nm in width, it was found that the change in the depth of the sheet at its center was ~ 0.5 Å over a 2000 K temperature variation range. We therefore believe that the adhesion energy obtained at 77 K will be closely comparable to that at 300 K.

Supplementary References

1. VandeVondele, J., Krack, M., Mohamed, F., Parrinello, M., Chassaing, T. & Hutter, J. QUICKSTEP: Fast and accurate density functional calculations using a mixed Gaussian and plane waves approach. *Comput. Phys. Commun.* **167**, 103-128 (2005).

2. Hutter, J., Iannuzzi, M., Schiffmann, F. & VandeVondele, J. CP2K: atomistic simulations of condensed matter systems. *Wiley Interdisciplinary Reviews-Computational Molecular Science,* **4** (1), 15-25 (2014).

3. VandeVondele, J. & Hutter, J. Gaussian basis sets for accurate calculations on molecular systems in gas and condensed phases. *J. Chem. Phys.* **127**, 114105 (2007).

4. Goedecker, S., Teter, M. & Hutter, J. Separable dual-space Gaussian pseudopotentials. *Phys. Rev. B* **54**, 1703-1710 (1996).

5. Perdew, J. P., Burke, K. & Ernzerhof, M. Generalized gradient approximation made simple. *Phys. Rev. Lett.* **77**, 3865-3868 (1996).

6. Grimme, S., Antony, J., Ehrlich, S. & Krieg, H. A consistent and accurate ab initio parametrization of density functional dispersion correction (DFT-D) for the 94 elements H-Pu. *J. Chem. Phys.* **132**, 154104 (2010).

7. Budrikis, Z. & Zapperi, S. Temperature-Dependent Adhesion of Graphene Suspended on a Trench. *Nano Lett.* **16,** 387–391 (2016).
